# Complete Genome Sequence of Porcine Epidemic Diarrhea Virus in Vietnam

**DOI:** 10.1128/genomeA.00753-14

**Published:** 2014-08-14

**Authors:** Dam Thi Vui, Nguyen Tung, Ken Inui, Steven Slater, Dachrit Nilubol

**Affiliations:** a Virology section, National Center for Veterinary Diagnosis, Department of Animal Health of Vietnam, Hanoi, Vietnam; b Epidemiology Division, Department of Animal Health of Vietnam, Hanoi, Vietnam; c Food and Agriculture Organization of the United Nations, Hanoi, Vietnam; d Great Lakes Bioenergy Research Center, the University of Wisconsin-Madison, Madison, Wisconsin, USA; e Department of Veterinary Microbiology, Faculty of Veterinary Science, Bangkok, Thailand

## Abstract

Porcine epidemic diarrhea virus (PEDV) has emerged in Vietnam since 2009. Herein, full-length genome sequences are reported for three PEDV isolates from pigs displaying severe diarrhea from farms located in northern and southern provinces of Vietnam. The results provide more understanding of the molecular characteristics of PEDV in Vietnam.

## GENOME ANNOUNCEMENT

Porcine epidemic diarrhea virus (PEDV), an enveloped single stranded positive-sense RNA virus in the genus *Alphacoronavirus*, family *Coronaviridae*, order *Nidovirales*, is a causative agent of porcine epidemic diarrhea (PED) (1), a disease characterized by severe watery diarrhea with high mortality in young pigs (2). PED is endemic in Asia (3–8), has been reported in Europe (2, 9), and its recent emergence in North America suggests a threat to the swine industry worldwide (10). PED has been observed in Vietnam since 2009, but no Vietnamese strains have previously been sequenced.

Intestinal samples were collected in Vietnam in 2013 from 3-day-old pigs displaying severe watery diarrhea. Three PEDV variants, designated VN/KCHY-310113, VAP1113_1, and JFP1013_1, were isolated using the vero cell line (11). Two came from swine farms in southern Vietnam and one came from the north. Total RNA was extracted from culture supernatant, and twelve overlapping regions of each genome were amplified, cloned in pGEM-T easy vector (Promega, USA), and sequenced in both directions in triplicate. The 5′ terminal sequences were determined by 5′ rapid amplification of cDNA ends (RACE) (12).

All three Vietnamese PEDV isolates are 28,035 nucleotides (nt) in length, and are greater than 99.8% and 99.6% identical at the nucleotide and amino acid levels, respectively. Genome organization resembles that of others PEDV genomes previously reported (1, 13), with gene order 5′-ORF1a/1b-S-ORF3-E-M-N-3'. The 5′ untranslated region (UTR) is 292 nt in length. The ORF1a/b gene is 20,344 nt in length. The sizes of the other six open reading frames, including S, ORF3, E, M, and N, are 4,158 nt, 675 nt, 231 nt, 681 nt, and 1,326 nt, respectively. The 3′ UTR is 334 nt in length.

A comparison to PEDV sequences available in GenBank demonstrated that the three new PEDV isolates share high similarity (98.6% to 98.7% and 97.7% to 98.0% at nucleotide and amino acid levels, respectively) with more recent isolates from China (LC, ZJCZ4, CHGD-01, CH/GDGZ/2012, GD-1, GD-A, and AJ1102) responsible for 2010–2012 outbreaks (14). All have unique characteristics including deletion and insertion in spike genes, which make them genetically distinct from CV777 and other earlier Chinese isolates (8). Specifically, spike genes in the Vietnamese strains have 2 insertions of 4 (^56^GENQ^59^) and 1 (^140^N) amino acids at amino acid positions 55 to 60 and 140, and 1 deletion of 2 (^160^DG^161^) amino acids at amino acid positions 160 to 161. The insertions and deletions are located in the hypervariable domain in the N-terminus of the S1 region.

This is the first report of full-length PEDV genomes from Vietnam. The full-length genome sequence suggests that PEDV variants circulating in Vietnam swine farms are novel variants with changes in the spike structure. The data provide valuable information on the molecular epidemiology of PEDV in Vietnam and will promote further investigation on genetic evolution and the selection of PEDV variants for vaccines that can be used to successfully control PED in Vietnam.

### Nucleotide sequence accession numbers.

The complete genome sequences of VN/KCHY-310113, VAP1113_1 and JFP1013_1 isolates have been deposited in GenBank under accession numbers KJ960178 through KJ960180.
